# Antibody response against PhoP efficiently discriminates among healthy individuals, tuberculosis patients and their contacts

**DOI:** 10.1371/journal.pone.0173769

**Published:** 2017-03-20

**Authors:** Aurobind Vidyarthi, Nargis Khan, Tapan Agnihotri, Kaneez F. Siddiqui, Girish R. Nair, Ashish Arora, Ashok K. Janmeja, Javed N. Agrewala

**Affiliations:** 1 CSIR-Institute of Microbial Technology, Chandigarh, India; 2 CSIR-Central Drug Research Institute, Lucknow, India; 3 Government Medical College and Hospital, Chandigarh, India; University of Cape Town, SOUTH AFRICA

## Abstract

Tuberculosis continues to be one of the most devastating global health problem. Its diagnosis will benefit in timely initiation of the treatment, cure and therefore reduction in the transmission of the disease. Tests are available, but none can be comprehensively relied on for its diagnosis; especially in TB-endemic zones. PhoP is a key player in *Mycobacterium tuberculosis* virulence but nothing has been known about its role in the diagnosis of TB. We monitored the presence of anti-PhoP antibodies in the healthy, patients and their contacts. In addition, we also measured antibodies against early secretory antigens ESAT-6 and CFP-10, and latency associated antigen Acr-1 to include proteins that are associated with the different stages of disease progression. Healthy subjects showed high antibody titer against PhoP than patients and their contacts. In addition, a distinct pattern in the ratio of Acr-1/PhoP was observed among all cohorts. This study for the first time demonstrates a novel role of anti-PhoP antibodies, as a possible marker for the diagnosis of TB and therefore will contribute in the appropriate action and management of the disease.

## Introduction

Tuberculosis (TB) patients produce antibodies to *Mycobacterium tuberculosis* (*Mtb)* proteins [1]. Considerable effort has been directed to understand the correlation between antibodies production and their specificity with disease progression[1–6]. Initial studies based on the response to purified protein derivatives (PPD) gives an indication of exposure to mycobacteria, but do not discriminate between *Bacillus-Calmette-Guerin* (BCG) vaccination and non-tuberculous mycobacteria (NTM) from *Mtb* infection[7, 8]. Consequently, it warrants the need of exploring novel tests for early diagnosis of TB.

Selection of suitable *Mtb* antigens for early diagnosis of TB is quite crucial. This should cover antigens secreted during early, latent and active form of infection. ESAT-6 is an early secreted low molecular weight antigen target. It can proficiently evoke both cell-mediated immunity and humoral immunity and therefore activates both T cells and B cells[9]. Culture filtrate protein (CFP)-10 is another antigen identified in the low-molecular-mass fraction of culture filtrate. The gene which encodes this antigen is located in the same operon as ESAT-6[10]. ESAT-6 and CFP-10 are not expressed in BCG and can therefore discriminate between BCG vaccinated and unvaccinated individuals. Thus, are potential antigens to test for the diagnosis of tuberculosis [11]. Many other antigens have also been considered important in regulating the virulence of *Mtb* [12].

Nearly one-third of the world population is infected with latent *Mtb*. Therefore, they are the major reservoir of the bacterium and possible threat of spreading TB. Alpha crystalline protein (Acr-1) is a 16 kDa Ag of *Mtb* that is predominantly expressed in the latent phase of infection[13]. PhoP is a part of PhoPR two-component system of *Mtb* and regulates key functions required for virulence and intracellular survival and persistence of *Mtb*[14]. PhoP is expressed in BCG also but very less is known about the pathways by which PhoP is activated [15, 16]. However, inactivation of PhoP results in down regulation of genes required for the existence of *Mtb* within the macrophages and consequently its attenuation. Accordingly, PhoP plays an important role in the virulence of the pathogen and thus represents a potential target for early diagnosis of TB[16]. Till date, nothing is reported concerning humoral response against PhoP in TB. Therefore, for the first time here we elucidated the novel role of PhoP in the diagnosis of TB. We document that PhoP showed a unique pattern of decreased antibody titer in TB patients and house-hold close contacts as compared to healthy individuals, which was not observed in any of the tested *Mtb* antigens *viz*. ESAT-6, CFP-10 and Acr-1. Further, we compared the ratio of antibodies generated against PhoP with early secretory antigens ESAT-6 and CFP-10, and latency Ag Acr-1. The change in the pattern of antibodies ratio of Acr-1/PhoP could discriminate between healthy individuals, TB patients and their house hold close contacts. These results signify the potential role of PhoP in the early diagnosis of TB.

## Material and methods

### Antigens used in the study

All the recombinant antigens, Acr-1 (heat shock protein hspX or 16 kDa or Rv2031c), CFP-10, ESAT-6 and PhoP were same, as used in the previous studies [13, 17].

### Patients and ethical clearance

The study was approved by the Institutional Ethics Committee, Government Medical College and Hospital, Chandigarh (No. 08776/4.2.10) and biosafety Institutional Biosafety Committee of the CSIR-Institute of Microbial Technology, Chandigarh, India (IMTECH/IBSC/2013/8) and experiments were performed in accordance with the ethical guidelines for biomedical research on human subjects by Central Ethics Committee on Human Research (CECHR), ICMR-2000. The blood was collected from Government Medical College and Hospital from the sputum positive pulmonary TB patients prior to initiating anti-tuberculosis therapy with informed written consents in accordance with the ethical guidelines for biomedical research on human subjects by Central Ethics Committee. The disease was clinically confirmed by the clinician who was expert in TB and chest diseases, by chest x-rays and Acid-fast bacilli smear positivity by microscopic examination. All the healthy subjects were from TB endemic zones, BCG vaccinated, but never had a past history of TB, and never lived with the patients diagnosed with active TB. Close contacts were considered those living with the patients diagnosed with active TB for minimum of six months. Further, Relapse were the TB patients who were cured using anti-tuberculosis therapy, but again diagnosed to be sputum smear positive. Diabetic patients correspond to sputum positive pulmonary TB patients and also diagnosed for diabetes. None of the cohorts in any of the three groups were positive for HIV. HIV was clinically confirmed through ELISA and western blot, diabetes was confirmed by blood sugar test by the clinician. S1 Table shows characteristics of healthy individuals, contacts and TB patients.

### ELISA

The standard ELISA protocol was followed for monitoring the presence of antibodies. Briefly, 96-well microtiter plates (BD Falcon, New Jersey, NJ) were coated with ESAT-6, CFP-10, Acr-1 and PhoP (50 μl of 5 μg/ml) in 0.2M carbonate bi-carbonate buffer (pH 9.6), overnight at 4°C. The plates were washed 3X with PBS-T20 (0.05%). Unsaturated sites of the wells were blocked with skimmed milk (5%) for 2h/37°C. The plates were washed 4x with PBS-T20. Different dilutions of serum, diluted in buffer (0.5% skimmed milk-0.025% Tween20-1XPBS) were poured in the plate and incubated for 1h/37°C. The plates were washed 5X and then incubated with goat anti-human IgG+A+M-HRP (STAR90P, AbD Serotec, Oxford, UK) diluted 1:10000 in PBS (1X) for 1h/37°C. The plates were washed 6X with PBST, and enzyme activity was assayed by incubation with 50 μl of O-phenylene diamine (P9029, P9029, Sigma-Aldrich, St. Louis, MO) (0.01%, wt/vol) and 30% H_2_O_2_ (0.01%, vol/vol)] diluted in PBS (1X) and incubated for 30 min/37°C. The reaction was stopped with 50 μl of H_2_SO_4_ (7%) and the optical density (OD) was measured at 492 nm.

### Blood

Blood samples (3-4 ml) of healthy volunteers, TB patients and their contacts were collected in vacutainers (BD, New Jersey, NJ). Within 1-2 h of collection, serum was separated by centrifugation at 1250g/7 min and stored in aliquots at −80°C, until use.

### Calculation of antibody titer

For calculating antibodies titer, cut off was determined by taking antigens negative control OD value (background) and adding arbitrarily 0.1 to it (to overcome background). Antibodies titre is the dilution factor of serum at which the OD of the sample is equal to the cut off OD.

### Statistics

All statistical calculations were conducted using graph pad prism. For comparison between groups, statistical analysis was done by using nonparametric tests and the Mann-Whitney test.

## Results

### Healthy volunteers, TB patients and their contacts show differential antibody response against PhoP

**Fig 1:**
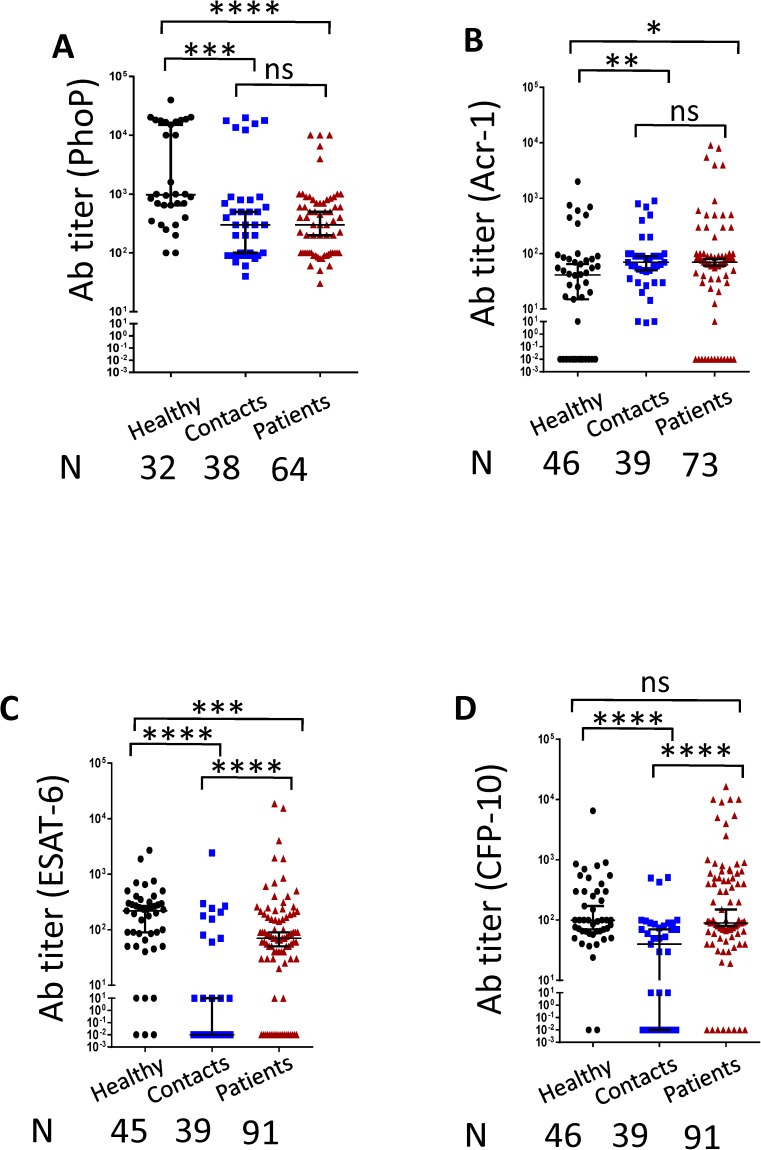
Compared to healthy subjects, TB patients display diminution in antibody levels against PhoP but not ESAT-6, CFP-10 and Acr1. Antibodies were measured in the serum of healthy, TB patients and close contacts against (A) PhoP; (B) Acr1; (C) ESAT-6 and (D) CFP-10. Median with 95% Cl represent the antibodies titers and each dot symbolizes single individual (N: number of individuals). *p<0.05, **p<0.01, ***p<0.001, ****p<0.0001, ns: non-significant.

**Fig 2:**
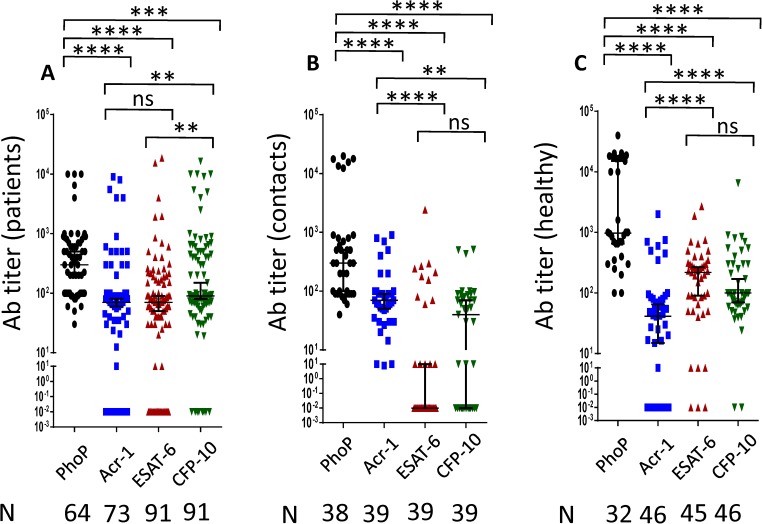
PhoP showed maximum antibody titer compared to ESAT-6, CFP-10 and Acr-1. Antibodies against PhoP, Acr1, ESAT-6 and CFP-10 were measured in the serum of (A) patients; (B) contacts; (C) healthy. Median with 95% Cl represent the antibodies titers and each dot symbolizes single individual (N: number of individuals). **p<0.01, ***p<0.001, ****p<0.0001, ns: non-significant.

PhoP is an important protein associated with the virulence of *Mtb*. Non-virulent strain of *Mtb* H37Ra upon expression of PhoP acquire virulence.[18]. Consequently, we thought that it would be imperative to monitor the antibodies response in the patients. We observed an interesting pattern in the antibody response against PhoP in TB patients, their contacts and healthy volunteers (Fig 1A). TB patients and contacts showed decrease in antibody titer than healthy individuals. As compared to healthy controls, the antibody titer was significantly decreased in the contacts (p<0.001) and TB patients (p<0.0001). In essence, the unique trend of down regulation of anti-PhoP antibodies in contacts and patients compared to healthy could not be observed in the other tested antigens (Fig 1A–1D). While comparing the response among the PhoP, Acr-1, ESAT-6 and CFP-10, it was observed that patients, contacts and healthy showed highest antibody titer against PhoP and this difference was highly significant as compared to Acr-1 and ESAT-6 (Fig 2). Patients exhibited highest titer against PhoP and followed by CFP-10 and least response to Acr-1 and ESAT-6 (Fig 2A). Similarly, contacts showed maximum level of anti-PhoP antibodies, followed by Acr-1 and least against ESAT-6 and CFP-10 (Fig 2B). Likewise, pattern of highest level of antibodies against PhoP were detected in healthy followed by ESAT-6, CFP-10 and least against Acr-1(Fig 2C). It is worth to mention here that the distinct pattern in the antibody response against PhoP in healthy volunteers, contacts and patients may provide information regarding the early diagnosis of TB.

### The ratio of antibodies titer for Acr-1 and PhoP discriminates between patients, close contacts and healthy individuals

**Fig 3:**
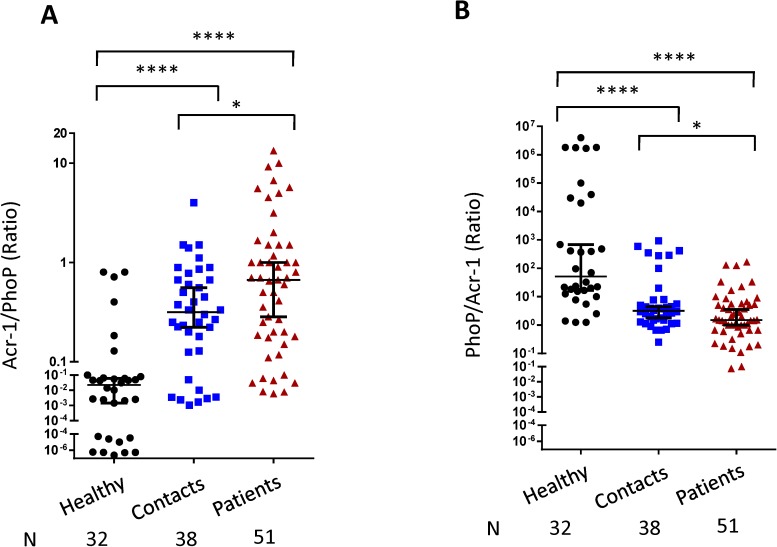
Ratio of Acr-1/PhoP antibody titer, discriminates among patients, contacts and healthy subjects. Antibodies ratio of (A) Acr-1/PhoP; (B) PhoP/Acr-1 were measured using the antibody titer against PhoP and Acr-1 in the serum of healthy, TB patients and close contacts. Median with 95% Cl represent the antibodies ratio between two antigens and each dot symbolizes single individual (N: number of individuals). *p<0.05, ****p<0.0001.

We next monitored the ratio between the tested antigens to get a better insight in understanding the antibody response to distinguish among patients, close contacts and healthy individuals. Intriguingly, based on the antibody ratio between Acr-1 and PhoP, it was noticed that patients exhibited significantly higher ratio than contacts (p<0.05), followed by the healthy subjects (p<0.0001) (Fig 3). No significant change in the pattern was revealed through PhoP/ESAT-6, PhoP/CFP-10, ESAT-6/PhoP, ESAT-6/CFP-10, ESAT-6/Acr-1, CFP-10/PhoP, CFP-10/ESAT-6, CFP-10/Acr-1, Acr-1/ESAT-6 and Acr-1/CFP-10 ratio (S1 Fig). Thus, it is evident from the results of Fig 3 that PhoP provides a unique pattern of antibody response that may differentiate patients, contacts and healthy from each other.

### Patients undergoing relapse exhibited suppressed antibody response against PhoP

**Fig 4:**
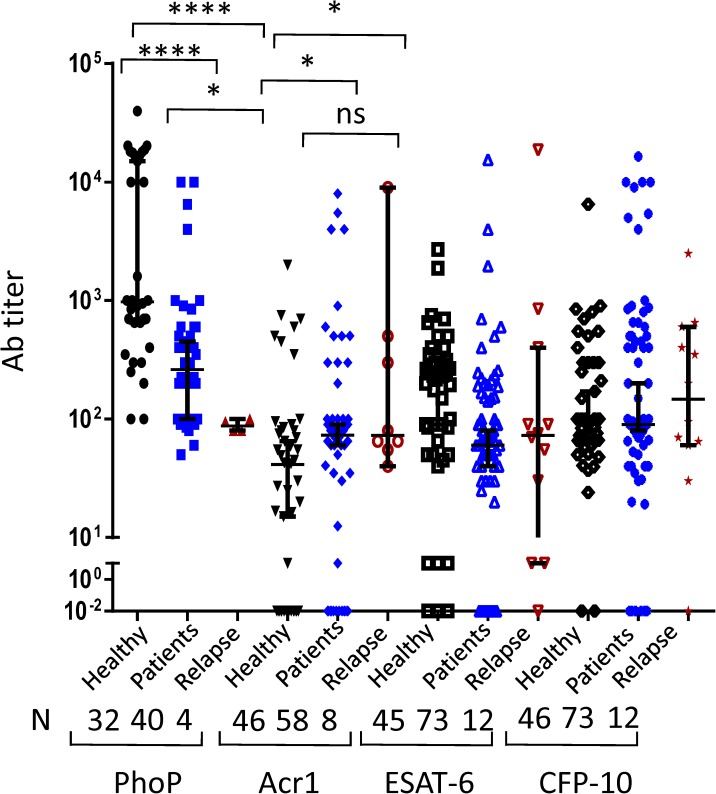
Relapsed cases show lower anti-PhoP antibodies than healthy volunteers. Antibodies against PhoP, Acr-1, ESAT-6 and CFP-10 were measured in the serum of healthy individuals, patients and relapsed cases. Median with 95% Cl represent the antibodies titers and each dot symbolizes single individual (N: number of individuals). *p<0.05, ****p<0.0001, ns: non-significant.

We were curious to know whether relapsed cases express differential antibody profile that could be a prognostic indicator of recurrence. Interestingly, relapsed patients showed significantly lower titer of anti-PhoP antibodies compared to patients (p<0.05) and maximum in healthy individuals (p<0.0001) (Fig 4). In contrast, highest antibody levels were observed in the patients against Acr-1 compared to healthy. No clear-cut trend was seen in the case of ESAT-6 and CFP-10. We also monitored the ratio of antibodies titer for Acr-1 and PhoP among relapse, patients and healthy individuals. It was observed that relapse patients exhibited higher ratio than patients and healthy subjects (S2 Fig). The patients used in this study were freshly diagnosed and were not under anti tuberculosis therapy. The suppression in the anti-PhoP antibodies and augmentation in anti-Acr1 antibodies may be an important indicator to predict the status of a person who may be in a state of undergoing relapse.

### TB patients suffering from diabetes demonstrate lower ESAT-6 antibody response compared to non-diabetic TB patients

**Fig 5:**
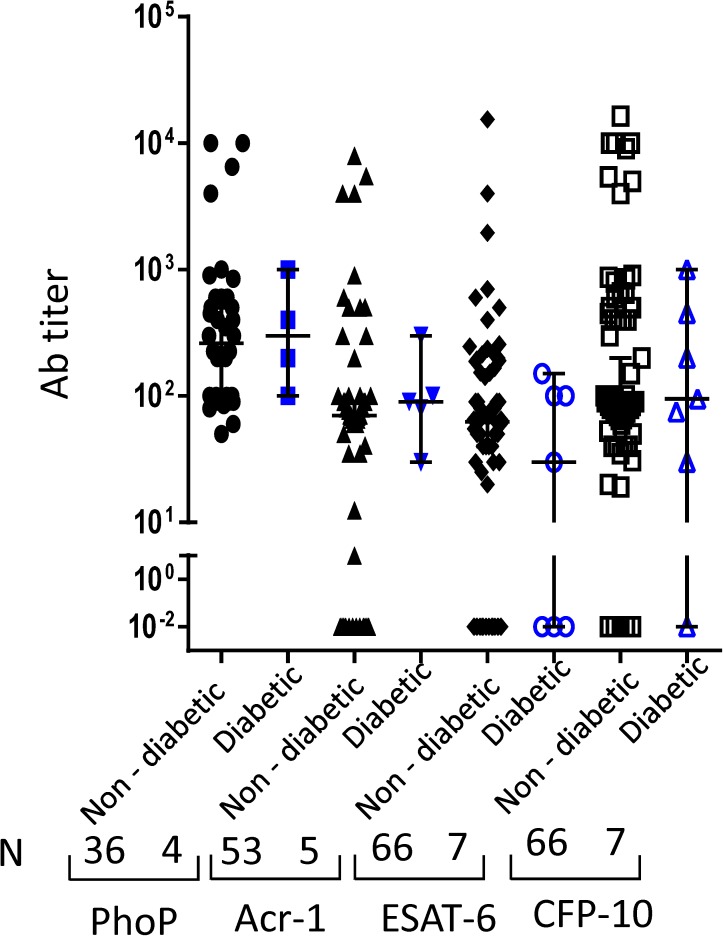
Diabetic TB patients show lower anti-ESAT-6 antibody response compared to non-diabetic TB patients. Antibodies against PhoP, Acr-1, ESAT-6 and CFP-10 were measured in the serum of diabetic TB patients and non-diabetic TB patients. Median with 95% Cl represent the antibodies titers and each dot symbolizes single individual (N: number of individuals).

We next thought to monitor the change in the antibody response between non-diabetics *versus* diabetic TB patients that could be of diagnostic importance. We observed that diabetic TB patients showed decline in antibodies against ESAT-6 (Fig 5). Further, we calculated antibody titer ratio of Acr1 and PhoP among diabetic and non-diabetic TB patients. It was noticed that diabetic patients showed increased Acr1/PhoP ratio as compared to non-diabetic patients (S3 Fig). The lower antibody response against ESAT-6 can be an indicator for diabetic patients predisposing towards TB.

## Discussion

TB is a leading infectious disease, responsible for about 2 million deaths annually[19]. Nine million people suffer from active disease and nearly one-third of the world population is latently infected with *Mtb*[20]. Latently infected people remain the largest reservoir of *Mtb* and a potential source of contracting active disease and its dissemination. Hence, it is extremely important to develop tests for early diagnosis of disease and therefore timely intervention can be provided for treatment and averting its propagation. Major knowledge for diagnosis of TB is derived through serodiagnostic studies [21–23]. Several reports have elucidated that the progression of TB to active disease may be predicted by elevated levels of *Mtb* specific antibodies[24]. Unfortunately, not much success was achieved probably due to failure of not employing appropriate antigens[25, 26].

PhoP is a unique protein, which is fundamental in maintaining the virulent character of *Mtb*[27]. Further, PhoP mutant strain is considered as a candidate vaccine against TB [28, 29]. Unfortunately, nothing has been reported concerning the role of immune response against PhoP in the TB patients or BCG vaccinated healthy subjects. Therefore, in the current study, we monitored the role of anti-PhoP antibodies generated in TB patients and whether their presence can be utilized for predicting the diagnosis of the disease. To further strengthen our study, we included early secretory antigens ESAT-6 and CFP-10 and latency associated protein Acr-1 to provide an insight into both the active and latent forms of the disease. Hence, in the current study, we monitored antibodies response against PhoP, Acr-1, ESAT-6 and CFP-10 in the healthy volunteers, TB patients and their close contacts and observed following interesting findings: i) unique pattern of antibody response against PhoP was observed with less in the case of TB patients and contacts and higher in healthy volunteers; ii) among the tested antigens, healthy subjects, TB patients and contacts showed maximum level of anti-PhoP antibodies; iii) highest antibody ratio of Acr-1/PhoP was noticed in patients followed by contacts and healthy subjects; iv) relapse cases illustrated decline in the anti-PhoP antibodies; v) TB patients suffering from diabetes exhibited lesser antibody response against ESAT-6 than the non-diabetic counterparts.

Among the tested antigens PhoP, Acr-1, ESAT-6 and CFP-10, reduction in the antibodies level against PhoP was quite prominent, irrespective of status of the TB patients i.e., whether they are relapsed. However, most striking and discrete finding of this study was that the healthy individuals from TB-endemic zones showed high level of antibodies against PhoP, whereas it considerably less in TB patients and contacts. Further, Acr1/PhoP ratio of antibodies was remarkably highest in patients, moderate in contacts and least in healthy subjects. It can be speculated from these results that the healthy individuals maintain optimum immune response against PhoP to protect them against developing TB. As the suppression in the yield of anti-PhoP antibodies occurs, the person is predisposed to TB. We conjecture that may be anti PhoP antibodies are protective against *Mtb*. Published reports also support the role of antibodies in defending from *Mtb*[30, 31]. Further, the reason behind higher anti PhoP antibodies in healthy individuals can be that PhoP is found in BCG and several Gram-negative species[15, 32]. It was also noted that the relapse cases also exhibited a remarkable drop in their anti-PhoP antibody titer. Thus, the follow-up studies for estimating the levels of anti-PhoP antibody and correlating the change may be quite crucial for the diagnosis of TB and its relapse.

We also demonstrated that diabetic TB patients showed lesser anti-ESAT-6 antibodies. This observation designates that as the severity of immunosuppression increases, the level of anti-ESAT-6 antibody decreases. Therefore, this may be an indicator of the severity of the disease.

In essence, the current study for the first time demonstrates a unique pattern of the Acr/PhoP ratio which can differentiate among healthy, contacts and TB patients. Further investigation is needed to develop a diagnostic test of TB using Acr/PhoP ratio. Intriguingly, this antibody ratio pattern could not be observed in the other tested antigens ESAT-6, Acr-1 and CFP-10. This observation will open a new avenue for an extensive follow-up studies to validate whether the drop in the level of anti-PhoP antibodies in healthy people render them susceptible to TB. Consequently, in future it may be advantageous for the early diagnosis of TB.

## Supporting information

S1 Fig Ratio of antibody titer among patients, contacts and healthy subjects. (PDF)

S2 Fig Ratio of Acr-1/PhoP antibody titer among relapse, patients and healthy subjects. (PDF)

S3 Fig Ratio of Acr-1/PhoP antibody titer among diabetic and non-diabetic TB patients. (PDF)

S1 Table Characteristics of healthy individuals, contacts and TB patients. (PDF)
