# Supplementary material for: Antibody response against PhoP efficiently discriminates among healthy individuals, tuberculosis patients and their contacts
Source: PLoS One. 2017 Mar 20;12(3):e0173769. doi: 10.1371/journal.pone.0173769 (PMC5358785; doi:10.1371/journal.pone.0173769)
Supplement: S1 Fig — (PDF) [file pone.0173769.s001.pdf]

S1 Fig

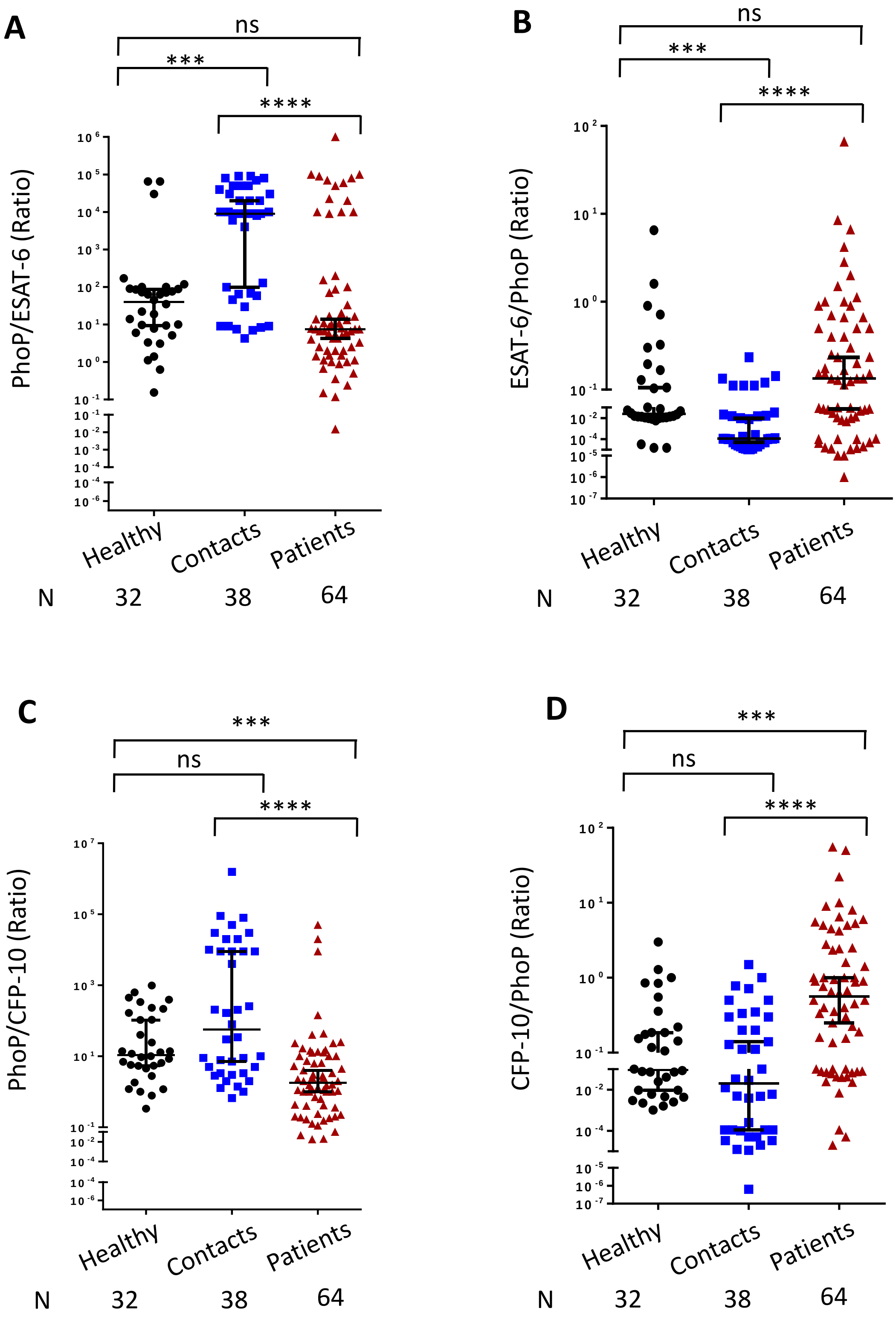

A
ns
***
****
PhoP/ESAT-6 (Ratio)
Healthy
Contacts
Patients
N
32
38
64
B
ns
***
****
ESAT-6/PhoP (Ratio)
Healthy
Contacts
Patients
N
32
38
64
C
***
ns
****
PhoP/CFP-10 (Ratio)
Healthy
Contacts
Patients
N
32
38
64
D
***
ns
****
CFP-10/PhoP (Ratio)
Healthy
Contacts
Patients
N
32
38
64

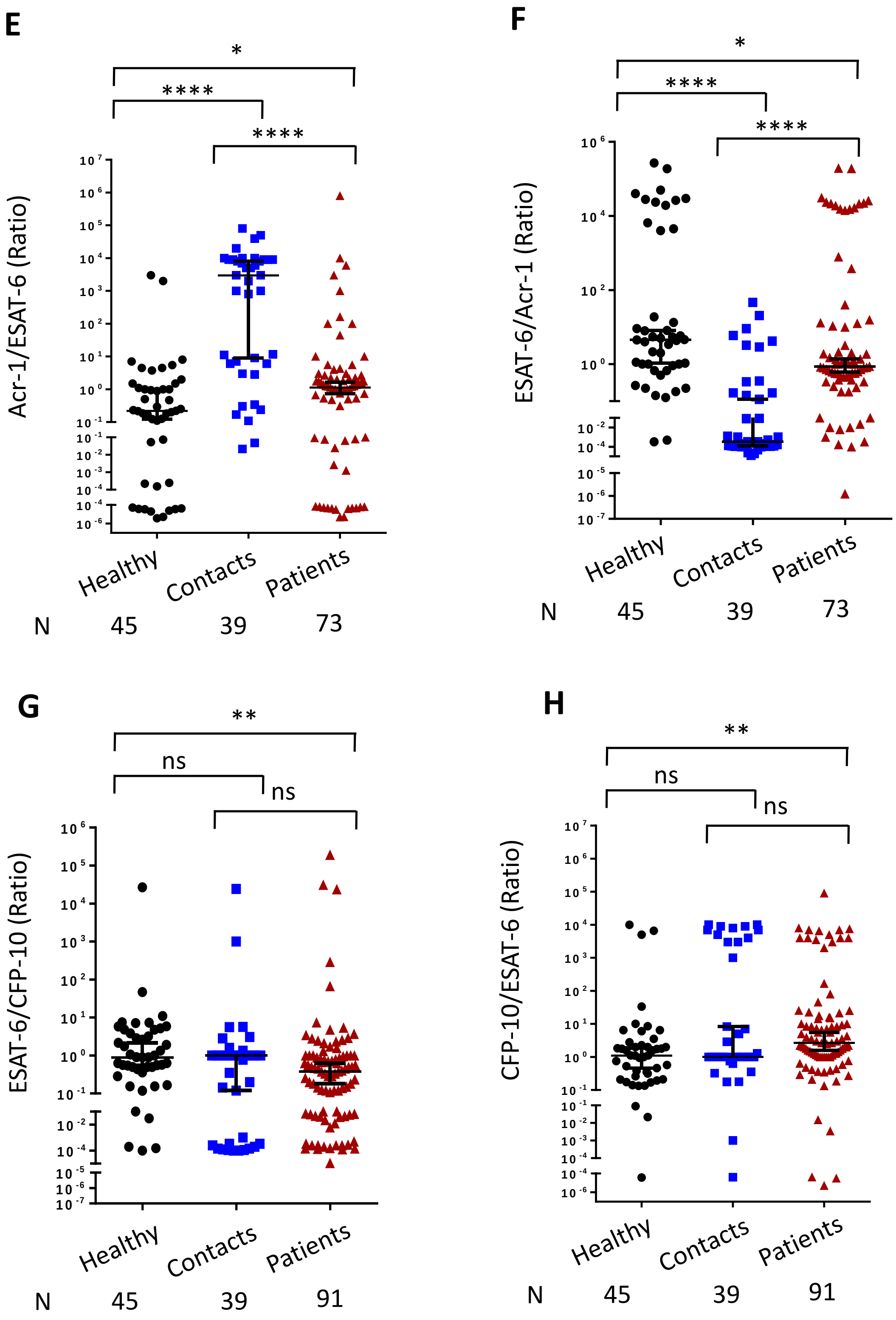
E
*
****
****
Acr-1/ESAT-6 (Ratio)
Healthy
Contacts
Patients
N
45
39
73
F
*
****
****
ESAT-6/Acr-1 (Ratio)
Healthy
Contacts
Patients
N
45
39
73
G
**
ns
ns
ESAT-6/CFP-10 (Ratio)
Healthy
Contacts
Patients
N
45
39
91
H
**
ns
ns
CFP-10/ESAT-6 (Ratio)
Healthy
Contacts
Patients
N
45
39
91

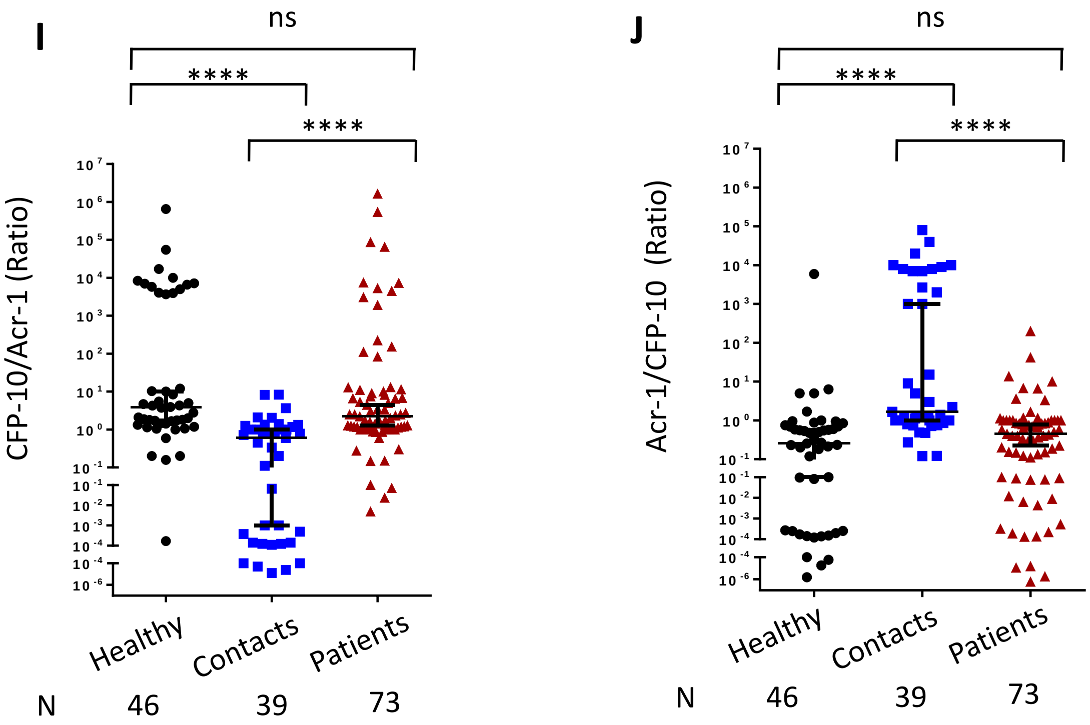

S1 Fig. Ratio of Antibody titer among patients, contacts and healthy subjects. Antibodies ratio (A) PhoP/ESAT-6; (B) ESAT-6/PhoP; (C) PhoP/CFP-10; (D) CFP-10/PhoP; (E) Acr-1/ESAT-6; (F) ESAT-6/Acr-1; (G) ESAT-6/CFP-10 (H) CFP-10/ESAT-6; (I) CFP-10/Acr-1; and (J) Acr-1/CFP-10 were measured using the antibody titer against PhoP, Acr-1, ESAT-6 and CFP-10 in the serum of healthy, TB patients and close contacts. Median with 95% Cl represent the Abs ratio between two Ags and each dot symbolizes single individual (N: number of individuals). *p<0.05, **p<0.01, ***p<0.001, ****p<0.0001.
