# Supplementary material for: Antibody response against PhoP efficiently discriminates among healthy individuals, tuberculosis patients and their contacts
Source: PLoS One. 2017 Mar 20;12(3):e0173769. doi: 10.1371/journal.pone.0173769 (PMC5358785; doi:10.1371/journal.pone.0173769)
Supplement: S2 Fig — (PDF) [file pone.0173769.s002.pdf]

S2 Fig

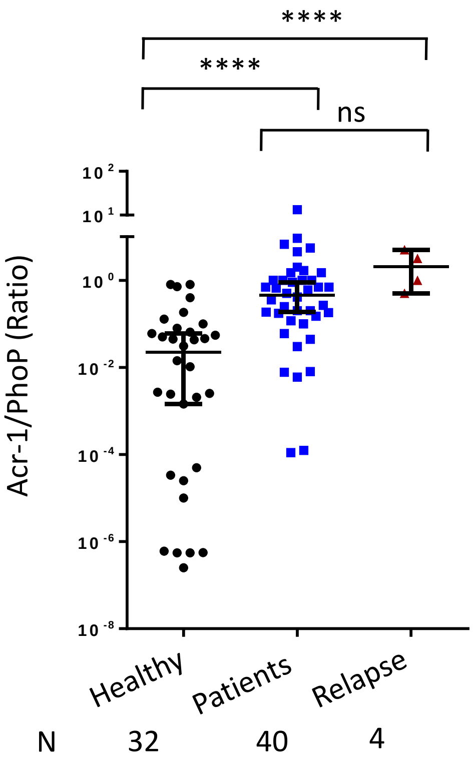

S2 Fig. Ratio of Acr-1/PhoP Antibody titer among relapse, patients and healthy subjects. Antibodies ratio were measured using the antibody titer against PhoP and Acr-1 in the serum of relapse, patients and healthy subjects. Median with 95% Cl represent the Abs ratio between two Ags and each dot symbolizes single individual (N: number of individuals). ns= non significant, ****p<0.0001.
