# Supplementary material for: Antibody response against PhoP efficiently discriminates among healthy individuals, tuberculosis patients and their contacts
Source: PLoS One. 2017 Mar 20;12(3):e0173769. doi: 10.1371/journal.pone.0173769 (PMC5358785; doi:10.1371/journal.pone.0173769)
Supplement: S3 Fig — (PDF) [file pone.0173769.s003.pdf]

S3 Fig

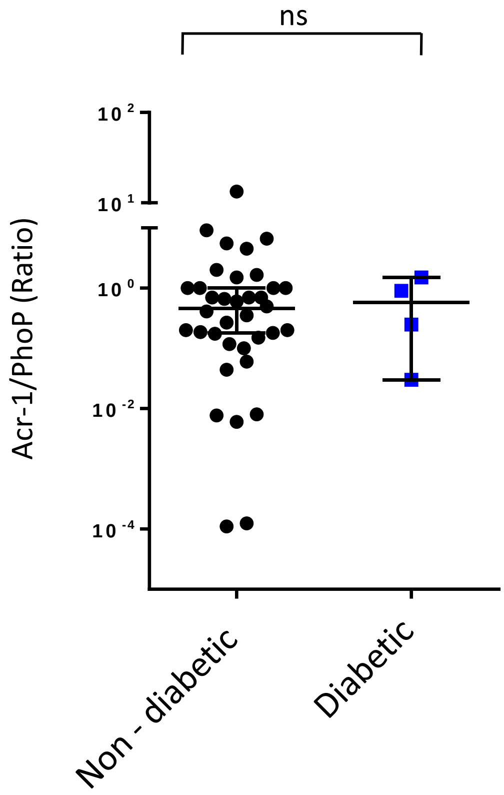

S3 Fig. Ratio of Acr-1/PhoP Antibody titer among diabetic and non-diabetic TB patients. Antibodies ratio were measured using the antibody titer against PhoP and Acr-1 in the serum of diabetic and non-diabetic TB patients. Median with 95% Cl represent the Abs ratio between two Ags and each dot symbolizes single individual (N: number of individuals). ns= non significant.
