# Supplementary material for: Antibody response against PhoP efficiently discriminates among healthy individuals, tuberculosis patients and their contacts
Source: PLoS One. 2017 Mar 20;12(3):e0173769. doi: 10.1371/journal.pone.0173769 (PMC5358785; doi:10.1371/journal.pone.0173769)
Supplement: S1 Table — (PDF) [file pone.0173769.s004.pdf]

S1 Table

Cohorts	Number of individuals	Sex, male Number (Percentage)	Age, years median (IQR)
**Healthy**<br>✓ BCG vaccinated<br>✓ Never had a past history of TB<br>✓ Never lived with a patient diagnosed with active TB	46	40 (86.9)	29 (27-32)
**Contacts**<br>✓ BCG vaccinated<br>✓ Never had a past history of TB<br>✓ Lived for minimum of six months with the patients diagnosed with active TB	39	22 (56.4)	34 (25-42.5)
**Patients**<br>✓ TB diagnosis was confirmed by chest x-rays and Acid-fast bacilli smear positivity	91	68 (74.7)	31 (21-48)
**Relapse**<br>✓ The TB patients who were cured using anti-tuberculosis therapy, but is again diagnosed to be sputum smear positive	12	11 (91.7)	41 (31-60)
**Diabetic**<br>✓ Sputum positive pulmonary TB patients also have diagnosed with diabetes	7	5 (71.4)	44 (21-52)

S1 Table. Characteristics of healthy individuals, contacts and TB patients. IQR: Interquartile range
